# Occurrence of virulence factors and antimicrobial susceptibility of *Citrobacter freundii* isolated from diseased ornamental fish in Poland

**DOI:** 10.2478/jvetres-2025-0017

**Published:** 2025-03-25

**Authors:** Anna Pastuszka, Leszek Guz, Krzysztof Puk, Dorota Pietras-Ożga

**Affiliations:** Department of Biology and Fish Diseases; Department of Epizootiology and Clinic of Infectious Diseases, Faculty of Veterinary Medicine, University of Life Sciences, 20-612 Lublin, Poland

**Keywords:** antibiotic resistance, aquarium environment, fish isolates

## Abstract

**Introduction:**

*Citrobacter freundii* strains, like other representatives of the same genus, are often described as aetiological factors of diseases in humans and animals, including farmed and ornamental fish. The aim of the study was the isolation and identification of *C. freundii* from ornamental fish in Poland and investigation of the virulence factors and antibiotic resistance among the isolates.

**Material and Methods:**

Twenty *C. freundii* isolates were isolated from swab samples obtained from diseased ornamental fish of 16 different species in Poland. The bacteria were initially identified to the species level by matrix-assisted laser desorption/ionisation time-of-flight mass spectrometry, and the results were confirmed based on sequencing of a fragment of the *16S rRNA* gene and biochemical profile analysis using the API-20E system. The antibiotic susceptibility profile was evaluated by the disc-diffusion method.

**Results:**

Most isolates were resistant to tetracycline (65%), ciprofloxacin (65%), nalidixic acid (60%) and doxycycline (50%). All of them were resistant to ampicillin. As many as 13/20 isolates (65%) carried the *viaB* gene encoding the Vi antigen and 9/20 (45%) carried the class 1 integron-encoding gene. The *hlyA* gene encoding the ability to produce α-haemolysins was not detected in any of the isolates tested.

**Conclusion:**

This is the first study to describe in detail the identification and occurrence of virulence factors and antibiotic resistance among *C. freundii* isolates of ornamental fish in Poland. The results indicate the need for further monitoring of the bacterium’s presence.

## Introduction

The genus *Citrobacter* comprises Gram-negative, facultative anaerobic, usually motile and non-sporeforming rod-shaped bacteria common in the environment (4). The first species of the genus was isolated in 1928 and classified as *Bacterium freundii*. Later, in 1932, it was named *Citrobacter freundii* (17). At present, according to the List of Prokaryotic names with Standing in Nomenclature (LPSN), 26 species in the *Citrobacter* genus have been reported (https://lpsn.dsmz.de/genus/citrobacter). *Citrobacter freundii* normally occurs as a commensal in the gastrointestinal tracts of both humans and animals (23). Nonetheless, partly for its ubiquity, it has been considered the most pathogenic opportunistic infectious agent in its genus, and is seen to cause various infections in humans, especially urinary tract infections, severe diarrhoea, wound infections, neonatal sepsis or brain abscesses in patients with meningitis (31, 35). There are also reports in the literature on the isolation of *C. freundii* from the tissues of diseased domestic and wild land animals, amphibians, reptiles, birds and aquatic animals, particularly farmed fish (4, 18, 41). Sato *et al*. (36) were the first to describe a case of isolation of *C. freundii* from infected sunfish (*Mola mola*). Cases of infections with this bacteria have been identified in other fish species, *e.g*. rainbow trout (*Oncorhynchus mykiss*) (10), Japanese eels (*Anguilla japonica*) (7) and catfish (species of the *Pseudoplatystoma* genus) (30). These bacteria are also widespread in the environment of ornamental fish (9, 12, 29, 42).

The main external predisposing factors for *C. freundii* infection are environmental stress, organic pollution, high water temperature and hypoxia (2). Clinical signs and pathological changes observed in diseased fish include haemorrhagic enteritis, haemorrhaging in the muscles and internal organs (kidneys, liver and gonads), erosion of the skin with loss of scales and diffuse bleeding on the skin and petechial haemorrhages on the gills, and these can lead to mass mortality (18, 30).

The major virulence genes found in the genome of *C. freundii* encode Shiga-like toxins, heat-stable toxins and haemolysins (6). *Citrobacter freundii* can also acquire the most common virulence factor found in *Salmonella enterica* serovars Typhi and Paratyphi, *i.e*. the capsular polysaccharide virulence antigen (Vi antigen) (22).

Clinical strains of *Citrobacter* spp. are resistant to various groups of antibiotics; however, the widespread use of these compounds in aquaculture has led to the emergence of resistance among isolates pathogenic to fish (15). Additionally, genes associated with bacterial resistance to antibiotics are often encoded on mobile genetic elements such as plasmids, integrons, insertion sequences and transposons, which significantly contribute to the dissemination of resistance and pathogenicity in horizontal gene transfer (33).

This study describes the first report of *C. freundii* infection of ornamental fish in Poland, including the biochemical profile, antimicrobial susceptibility and results of genetic tests, with the aim of better understanding the virulence of these isolates.

## Material and Methods

### Collection and maintenance of bacterial isolates

*Citrobacter* spp. isolates (n = 20) were obtained from diseased ornamental fish referred for testing to the Department of Biology and Fish Diseases in the Faculty of Veterinary Medicine of the University of Life Sciences in Lublin in the period from September 2022 to June 2023. The bacteria were isolated from 16 different species of fish (Table 1). The fish were euthanised with MS-222 (tricaine methanesulphonate, Sigma-Aldrich, St. Louis, MO, USA) at a dose of 200 mg l^−1^. Swabs for bacteriological tests were taken from the skin, trunk kidney, liver and other diseased sites (ulcers or frayed fins, if observed). They were inoculated onto tryptic soy agar (TSA, Oxoid, Basingstoke, UK) and incubated at 36°C for 24–48 h. Then the bacterial colonies were carefully examined. Quantitatively dominant and morphologically similar colonies were transferred to fresh TSA plates to obtain pure cultures. These cultures were stored at –80°C in the Microbank system (Pro-Lab Diagnostics, Bromborough, UK) for further identification.

**Table 1.** Origin and identification by matrix-assisted laser desorption/ionisation–time-of-flight mass spectrometry (MALDI-TOF MS) of *Citrobacter freundii* strains isolated from ornamental fish in Poland

Strain signature	Host species	Habitat / year of sampling	Source of isolate	Clinical signs of disease	Presence of other pathogens in the material	MALDI-TOF MS	
						Score values	Species ID (based on MALDI Biotyper 3.1 software)
*Citrobacter freundii*_1	*Trichopodus trichopterus*	Aquatic env. /2023	Trunk kidney	Lethargy, debilitated condition, no appetite, haemorrhages on the fin bases and around the anus and mouth	*Aeromonas hydrophila*	2.367	*C. freundii* 22054_1 CHB
*Citrobacter freundii*_2	*Poecilia wingei*	Aquatic env. / 2023	Trunk kidney	Lethargy, gill necrosis, increased respiration, septicaemia	*Acinetobacter lwoffii*	2.037	*C. freundii* 22054_1 CHB
*Citrobacter freundii*_3	*Tanichthys albonubes*	Aquatic env. / 2023	Skin ulcers	Haemorrhagic ulcerations in the skin, haemorrhages around the anus	-	2.236	*C. freundii* 22054_1 CHB
*Citrobacter freundii*_4	*Xiphophorus maculatus*	Aquatic env. / 2022	Liver	Ocular opacity, exophthalmos, abdominal distention	-	2.151	*C. freundii* 22054_1 CHB
*Citrobacter freundii*_5	*Corydoras paleatus*	Aquatic env. / 2023	Skin ulcers	Haemorrhagic ulcerations in the skin, erratic swimming, no appetite	*Aeromonas veronii*	2.225	*C. freundii* 13158_2 CHB
*Citrobacter freundii*_6	*Hypostomus plecostomus*	Aquatic env. / 2022	Liver	Weakened condition, fluid accumulation in the abdominal cavity	*Aeromonas caviae, Vibrio albensis*	2.211	*C. freundii* 13158_2 CHB
*Citrobacter freundii*_7	*Sahyadria denisonii*	Aquatic env. / 2023	Skin ulcers	Haemorrhagic ulcerations in the skin, haemorrhages on the fin bases and around the anus and mouth	-	2.112	*C. freundii* DSM 30039T HAM
*Citrobacter freundii*_8	*Chromobotia macracanthus*	Aquatic env. / 2022	Trunk kidney	No appetite, swimming disorders, caudal fin with frayed edges	*Pseudomonas* *alcaligenes*	2.284	*C. freundii* DSM 15979 DSM
*Citrobacter freundii*_9	*Xiphophorus maculatus*	Aquatic env. / 2023	Liver	Lethargy, swimming disorders, caudal fin with frayed edges, haemorrhages on the fin bases	*Plesiomonas shigelloides*	2.216	*C. freundii* 22054_1 CHB
*Citrobacter* *freundii*_10	*Betta splendens*	Aquatic env. / 2022	Skin ulcers	Haemorrhagic ulcerations in the skin	*Shewanella* *putrefaciens*	2.268	*C. freundii* 22054_1 CHB
*Citrobacter* *freundii*_11	*Poecilia sphenops*	Aquatic env. / 2023	Trunk kidney	Emaciation, lethargy, haemorrhages around the anus and mouth	*Pseudomonas putida*	2.340	*C. freundii* 22054_1 CHB
*Citrobacter* *freundii*_12	*Betta splendens*	Aquatic env. / 2022	Trunk kidney	Swimming disorders, exophthalmos, fluid accumulation in the abdominal cavity	*Aeromonas veronii, Shewanella putrefaciens*	2.402	*C. freundii* 13158_2 CHB
*Citrobacter* *freundii*_13	*Hemigrammus rhodostomus*	Aquatic env. / 2023	Skin ulcers	Haemorrhagic ulcerations in the skin	-	2.059	*C. freundii* 13158_2 CHB
*Citrobacter* *freundii*_14	*Betta splendens*	Aquatic env. / 2023	Trunk kidney	Weakened condition, caudal fin with frayed edges	-	2.229	*C. freundii* 22054_1 CHB
*Citrobacter* *freundii*_15	*Pseudotropheus Saulosi*	Aquatic env. / 2023	Liver	Haemorrhaging in the eye and exophthalmia, abdominal distention	*Aeromonas hydrophila*	2.094	*C. freundii* 13158_2 CHB
*Citrobacter* *freundii*_16	*Xiphophorus hellerii*	Aquatic env. / 2022	Skin ulcers	Skin discolouration, haemorrhagic spots and superficial ulcers	-	2.253	*C. freundii* 22054_1 CHB
*Citrobacter* *freundii*_17	*Macropodus opercularis*	Aquatic env. / 2022	Liver	Haemorrhages on the fin bases and around the anus and mouth	-	2.300	*C. freundii* 22054_1 CHB
*Citrobacter* *freundii*_18	*Danio rerio*	Aquatic env. / 2022	Liver	Skin discoloration, haemorrhagic spots, fluid accumulation in the abdominal cavity	*Chryseobacterium indologenes*	2.254	*C. freundii* 22054_1 CHB
*Citrobacter* *freundii*_19	*Mikrogeophagus ramirezi*	Aquatic env. / 2022	Liver	Weakened condition, no appetite, petechial haemorrhaging in the liver and intestines	*Aeromonas veronii, Pseudomonas putida*	2.378	*C. freundii* 22054_1 CHB
*Citrobacter* *freundii*_20	*Mikrogeophagus ramirezi*	Aqu. Env / 2023	Liver	Lethargy, debilitated condition, no appetite, increased respiration, hemorrhages on the fin bases	-	2.049	*C. freundii* 13158_2 CHB

### Identification by matrix-assisted laser desorption/ionisation-time-of-flight mass spectrometry (MALDI-TOF-MS)

Phenotypic identification was based on analysis of the proteomic profile of isolates using the MALDI-TOF-MS technique (32). A bacterial assay standard containing a typical protein extract of *Escherichia coli* DH5α (Bruker Daltonics, Bremen, Germany) was used to calibrate the mass spectrometer. Following taxonomic identification of the microorganisms, a MALDI dendrogram was prepared using tools included in MALDI Biotyper 3.1 software (Bruker Daltonics) to determine the relationships between isolates. Mass spectral profiles (MSPs) were generated based on the mass spectra obtained for each strain, taking all masses present in the spectra and their intensities into account. Standard settings were used for matching and grouping: the maximum mass error tolerance for each spectrum was 2,000 mg L^−1^, the required mass error for mass spectral profiles was 200 mg L^−1^, the required minimum peak frequency was 25% and the maximum required number of peaks was 70. The relationships between individual MSPs were illustrated using the unweighted pair group with arithmetic mean (UPGMA) method.

### Biochemical and morphological examination

Gram staining of all 20 *Citrobacter* spp. isolates was performed using a standard protocol (5). In addition, an oxidase test was performed using a commercial oxidase reagent (bioMérieux, Marcy-l’Étoile, France). The motility of the bacteria was tested in semi-liquid lysogeny broth medium (Invitrogen/Thermo Fisher Scientific, Carlsbad, CA, USA) with the addition of 0.3% bacteriological lab agar (BioMaxima, Lublin, Poland). The motility zones were analysed after 24 h of incubation at 30°C. Blood-agar plates (BD Difco, Detroit, MI, USA) were used to test the capacity of *Citrobacter* spp. bacteria to produce haemolysis. The inoculated plates were incubated at 37°C for 24 h. To test the preferences of the isolates for lactose utilisation, they were cultured on standard MacConkey medium (MacConkey LAB-AGAR, BioMaxima) at 37°C for 24 h. Biochemical identification was performed using commercial API-20E kits (bioMérieux), with *Escherichia coli* American Tissue Culture Collection (ATCC) 25922 as the standard strain.

### Antibiotic susceptibility test

The antibiotic susceptibility profiles of the *Citrobacter* spp. isolates were tested by the Kirby–Bauer disc diffusion method (3) using Mueller–Hinton agar plates (Sigma-Aldrich) and commercial discs with antibiotics (BioMaxima and Oxoid). The effects of the following drugs in the classes shown in Supplementary Table 1 were assessed: ampicillin (used at 10 μg), piperacillin-tazobactam (TZP, 110 μg), cefotaxime (CTX, 30 μg), imipenem (IPM, 10 μg), meropenem (MEM, 10 μg), gentamicin (CN, 10 μg), amikacin (AK, 30 μg), kanamycin (used at 30 μg), streptomycin (used at 10 μg), tetracycline (TE, 30 μg), doxycycline (DO, 30 μg), ciprofloxacin (CIP, 5 μg), nalidixic acid (NA, 30 μg), sulfamethoxazole-trimethoprim (used at 25 μg) and chloramphenicol (used at 30 μg). The test was carried out in accordance with the Clinical and Laboratory Standards Institute guidelines for Enterobacterales in CLSI document M100 (8). The plates were incubated at 35°C for 18 h. After this time, the diameter of the inhibition zones was compared with the standard. The *Citrobacter* spp. isolates were characterised as susceptible, intermediately susceptible or resistant to the antibiotics based on the CLSI guidelines and those listed in Table 2.

**Table 2.** Antibiotic resistance profiles for all tested *Citrobacter* isolates from freshwater and ornamental fish (n = 20)

Antibiotic	Bacterial isolates’ antibiogram profile and interpretative range for disc diffusion					
	Resistant (%)	Zone diameter (mm)	Intermediate (%)	Zone diameter (mm)	Susceptible (%)	Zone diameter (mm)
Ampicillin (AM10 μg)	20(100)	≤13	0 (0)	14–16	0 (0)	≥17
Piperacillin-tazobactam (TZP 110 μg)	0 (0)	≤17	1 (5)	18–20	19 (95)	≥21
Cefotaxime (CTX 30 μg)	2 (10)	≤22	7 (35)	23–25	11 (55)	≥26
Imipenem (IPM 10 μg)	1 (5)	≤19	1 (5)	20–22	18 (90)	≥23
Meropenem (MEM 10 μg)	0 (0)	≤19	1 (5)	20–22	19 (95)	≥23
Gentamicin (CN 10 μg)	0 (0)	≤12	1 (5)	13–14	19 (95)	≥15
Amikacin (AK 30 μg)	2 (10)	≤14	3 (15)	15–16	15 (75)	≥17
Kanamycin (K 30 μg)	5 (25)	≤16	9 (45)	14–17	6 (30)	≥18
Streptomycin (S 10 μg)	9 (45)	≤11	8 (40)	12–14	3 (15)	≥15
Tetracycline (TE 30 μg)	12 (60)	≤11	0 (0)	12–14	8 (40)	≥15
Doxycycline (DO 30 μg)	10 (50)	≤10	3 (15)	11–13	7 (35)	≥14
Ciprofloxacin (CIP 5 μg)	13 (65)	≤21	6 (30)	22–25	1 (5)	≥26
Nalidixic acid (NA 30 μg)	12 (60)	≤13	3 (15)	14–18	5 (25)	≥19
Sulfamethoxazole-trimethoprim (SXT 25 μg)	8 (40)	≤10	0 (0)	11–15	12 (60)	≥16
Chloramphenicol (C30 μg)	9 (45)	≤12	0 (0)	13–17	11 (55)	≥18

The multiple antimicrobial resistance index (MAR) was calculated for each isolate (21). *Escherichia coli* ATCC 25922 and *Pseudomonas aeruginosa* ATCC 27853 (for carbapenems) were used as quality control strains.

### Genomic DNA isolation and PCR amplification

DNA was extracted from an overnight culture obtained in tryptic soy broth at 37°C with a Bacterial and Yeast Genomic DNA Purification Kit (EURx, Gdańsk, Poland) used according to the manufacturer’s recommendations. The oligonucleotide primers for the amplification sequence of partial *16S rRNA* have previously been described, and were (as the forward primer) 27F (5′ AGA GTT TGA TCM TGG CTC AG 3′) and (as the reverse primer) 1492R (5′ TAC GGY TAC CTT GTT ACG AC TT 3′) (14). The reaction conditions and the composition of the reaction mixture published in existing protocols were adopted (14). A Gel-Out kit (A&A Biotechnology, Gdańsk, Poland) was used to extract and purify the PCR products. The amplicons were subjected to the Sanger sequencing method (Genomed, Warsaw, Poland), performed with a forward and reverse primer for PCR. The sequences obtained and their nearest matches were selected for further phylogenetic analysis, using ClustalW (40) as a tool to align the sequences and MEGA version 11 (39) for cluster analysis. A phylogenetic tree was created using the neighbour-joining method with 500 bootstrap replicates, in which the evolutionary distances were computed using the maximum-composite-likelihood algorithm.

### PCR screening for known *Citrobacter freundii* virulence and antibiotic resistance genes

The primer sequences were synthesised by Genomed. The presence of four *C. freundii* virulence factors and three tetracycline resistance genes was investigated. Detailed information on PCR product size, primer sequences and temperature of amplification is summarised in Table 3. Each of the amplification reactions described above was performed in a GeneExplorer GE-96G Thermal Cycler (Bioer Technology, Hangzhou, China), and the products were subjected to electrophoretic separation (120V) on an agarose gel (1.5%) stained with Simply Safe (EURx).

**Table 3.** Primers used for virulence and antibiotic resistance gene expression study in *Citrobacter freundii* isolates from ornamental fish in Poland

Primer name	Primer sequence (5′–3′)	Target	Temperature of annealing	Amplicon size (bp)	Reference
viaB	F-TGTCGAGCAGATGGATGAGCAT R-ACGGCTGAAGGTTACGGACCGA	Vi capsular polysaccharide gene (Vi antigen)	62°C	516	14
LT	F-ACGGCGTTACTATCCTCTC R-TGGTCTCGGTCAGATATGTG	Heat-labile toxin gene of *Escherichia coli*	62°C	273	38
STp	F-TCTTTCCCCTCTTTTAGTCAG R-ACAGGCAGGATTACAACAAAG	Heat-stable toxin gene of *Escherichia coli*	62°C	166	38
hlyA	F-GTCTGCAAAGCAATCCGCTGCAAATAAA R-CTGTGTCCACGAGTTGGTTGATTAG	α-haemolysin gene	58°C	561	19
hep 58–59	F-TCATGGCTTGTTATGACTGT R-GTAGGGCTTATTATGCACGC	Class 1 integron-encoding gene	55°	200	44
tetA	F-CGCYTATATYGCCGAYATCAC R-CCRAAWKCGGCWAGCGA	Tetracycline resistance genes (tetracycline efflux pumps)	60°	417	26
tetB	F-GGDATTGGBCTTATYATGCC R-ATMACKCCCTGYAATGCA		60%	967	26
tetG	F-TATGCRTTKATGCAGGTC R-GACRAKCCAAACCCAACC		60%	917	26

1 F – forward; R – reverse

## Results

### Bacterial isolation and preliminary identification

Detailed information about the origin of the strains is presented in Table 1. Among the bacterial samples isolated from ornamental fish and initially identified based on the MALDI-TOF MS method, 20 strains belonging to the species *Citrobacter freundii* were selected and numbered (1 to 20). Twenty amplicons of approximately 1,350 base pairs were obtained from these isolates. Most bacterial strains had MALDI-TOF scores no lower than 2.000, which guaranteed secure genus and probable species identification (*C. freundii* _02, 03, 04, 05, 06, 07, 08, 09, 10, 13, 14, 15, 16, 18 and 20). In the case of strains 01 (from *Trichopodus trichopterus*), 11 (from *Poecilia sphenops*), 12 (from *Betta splendens*), 17 (from *Macropodus opercularis*) and 19 (from *Mikrogeophagus ramirezi*), the score was 2.300 or above, which confirmed secure species identification (Table 1).

**Fig. 1.**
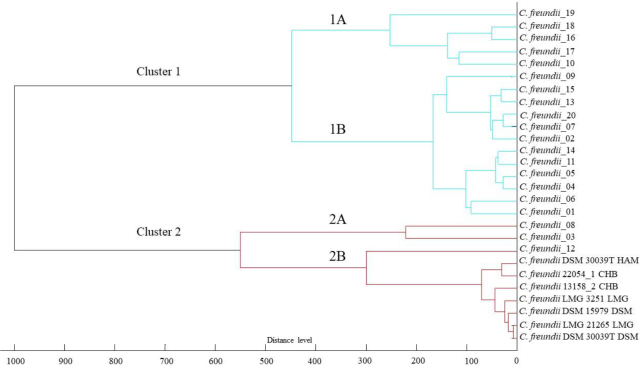
Phyloproteomic tree of *Citrobacter freundii* isolates from ornamental fish and reference strains, obtained on the basis of protein profile analysis and mass spectral profile identification using the MALDI (matrix-assisted laser desorption/ionisation) Biotyper platform

The similarity among the *C. freundii* strains based on their MALDI-TOF mass spectra is illustrated in the phyloproteomic dendrogram derived from UPGMA cluster analysis (Fig. 1). Two clusters formed this dendrogram made of the 20 *C. freundii* isolates selected from diseased ornamental fish and seven standard strains whose mass spectra were available in the reference database (*C. freundii* 22054_1 CHB, *C. freundii* 13158_2 CHB, *C. freundii* LMG21265 LMG, *C. freundii* LMG3251 LMG, *C. freundii* DSM30039T HAM, *C. freundii* DSM15979 DSM and *C. freundii* DSM30039T DSM) (Fig. 1). The first is larger and contains most of the isolates with 17/20 (85%). It is further divided into two branches: one (1A) bears only 5/20 (25%) of the *C. freundii* isolates, while the other (1B) holds the remaining 12/20 (60%) isolates of this cluster (Fig. 1). The second cluster is also divided into two branches (2A and 2B), the latter containing 1/20 (15%) of the tested isolates (*C. freundii*_12) and all reference strains, and the former carrying two closely related strains, *C. freundii*_08 and *C. freundii*_03.

### Morphological and biochemical tests

The microscopic observations and preliminary biochemical examination revealed that all these isolates were Gram-negative, rod-shaped, oxidase-negative strains (Supplementary Table 2). Nineteen of them were capable of flagellum-dependent movement in a platebased swimming motility test in semi-liquid lysogeny broth containing 0.3% agar. Migration zone diameters ranged from 30 to 71 mm after 24-h incubation (Supplementary Table 2). *Citrobacter freundii*_08 only migrated 8 mm, which means that the bacteria grew at the inoculation point and the strain was not motile. All 20 *C. freundii* strains were non-haemolytic, displaying γ-haemolysis (Supplementary Table 2). The *C. freundii* colonies grew on MacConkey ferment lactose (Supplementary Table 2).

Commercial API-20E tests were used to broadly identify the features of bacterial metabolism. On this basis, all strains were biochemically identified as *C. freundii* (Supplementary Table 2). The percentage probability of identification for 16 isolates (*C.f*_01, 03, 05, 07, 11, 13, 15, 16, 17, 18, 19 and 20) was very high, amounting to over 99% (Supplementary Table 2).

### Antimicrobial susceptibility testing

Most of the isolates were resistant to tetracyclines (12/20 to TE and 10/20 to DO) and quinolones (13/20 to CIP and 12/20 to NA) (Supplementary Table 1 and Table 2). All 20 (100%) were resistant to ampicillin; according to the CLSI (8), *Citrobacter* spp. isolates have intrinsic resistance to ampicillin, and this study’s confirmation of this was expected (Table 2). By contrast, majorities of the strains showed susceptibility to TZP, 19/20 doing so (95%); to CTX, 11/20 failing to resist it (55%); to CN, which was effective against 19/20 (95%); and to AK, 15/20 proving susceptible (75%), as shown in Table 2. Carbapenems also proved effective: only one strain was resistant and one intermediately susceptible to IPM (90% were susceptible), and only one strain was intermediately susceptible to MEM (95% were susceptible) (Supplementary Table 1 and Table 2). The highest MAR index among *Citrobacter* spp. bacteria was observed for *C. freundii*_12 and was 0.67, which means that the strain was resistant to 10 of 15 agents used (Supplementary Table 1). In addition to this strain, the *Cf*_01, *Cf*_04, *Cf*_05, *Cf*_06, *Cf*_08, *Cf*_10, *Cf*_18 and *Cf*_19 isolates could be classified as multidrug-resistant (MDR) bacteria, as they were resistant to three or more classes of antibiotics used (Supplementary Table 1).

### Genetic identification and phylogenetic analysis

Following amplification and *16S rRNA* sequencing of all *Citrobacter* spp. isolates, the sequences showed a percentage of identity ranging from 98.51% to 99.85% to those of reference strains of *C. freundii* logged in the GenBank nucleotide sequence database (Table 4). The sequences were subsequently deposited in GenBank under accession Nos PP264335–PP264354.

**Table 4.** Basic local alignment search tool results of sequence analysis of *16S rRNA* amplicons and genotypic profiles of *Citrobacter freundii* (n = 20) isolates from ornamental fish in Poland

Isolate	Length of *16S rRNA* sequence deposited in GenBank	Accession number	Percentage identity	Sequence name/ID (GenBank)	Presence of virulence genes (viaB/hlyA/LT/STp primers)	Presence of Class 1 integron-encoding gene (hep58–59 primers)	Presence of tetracycline resistance genes (tetA/tetB/tetG primers)
*C.f_*01	1,348 bp	PP264335	99.41%	*C. freundii* ATCC 8090/NR_028894.1	+/–/–/–	+	–/+/+
*C. f_*02	1,353 bp	PP264336	99.11%	*C. freundii* ATCC 8090/NR_028894.1	+/–/–/–	–	–/–/–
*C. f_*03	1,348 bp	PP264337	99.63%	*C. freundii* strain LMG 3246/NR_117752.1	–/–/+/–	–	–/–/–
*C. f_*04	1,349 bp	PP264338	99.26%	*C. freundii* ATCC 8090/NR_028894.1	+/–/–/+	+	–/–/–
*C. f_*05	1,352 bp	PP264339	99.70%	*C. freundii* ATCC 8090/NR_028894.1	–/–/–/+	+	+/+/+
*C. f_*06	1,349 bp	PP264340	99.78%	*C. freundii* ATCC 8090/NR_028894	*+*/–/–/–	+	+/–/+
*C. f_*07	1,351 bp	PP264341	99.85%	*C. freundii* strain LMG 3246/NR_117752.1	–/–/–/–	–	–/–/–
*C. f_*08	1,347 bp	PP264342	99.41%	*C. freundii* ATCC 8090/NR_028894.1	*+*/–/–/–	+	+/–/+
*C. f_*09	1,346 bp	PP264343	99.24%	*C. freundii* strain NBRC 12681/NR_114345.1	*+*/–/–/–	–	–/–/+
*C. f_*10	1,354 bp	PP264344	99.34%	*C. freundii* ATCC 8090/NR_028894.1	*+*/–/–/–	+	+/–/+
*C. f_*11	1,349 bp	PP264345	99.56%	*C. freundii* ATCC 8090/NR_028894.1	*+*/–/–/–	–	+/–/+
*C. f_*12	1,351 bp	PP264346	99.41%	*C. freundii* ATCC 8090/NR_028894.1	*+*/–/–/–	+	+/–/+
*C. f_*13	1,352 bp	PP264347	99.63%	*C. freundii* ATCC 8090/NR_028894.1	–/–/–/–	–	–/–/–
*C. f_*14	1,351 bp	PP264348	99.56%	*C. freundii* strain LMG 3246/NR_117752.1	*+*/–/–/–	–	–/–/–
*C. f_*15	1,351 bp	PP264349	99.78%	*C. freundii* ATCC 8090/NR_028894.1	–/–/–/–	–	–/–/–
*C. f_*16	1,352 bp	PP264350	99.56%	*C. freundii* strain LMG 3246/NR_117752.1	–/–/–/–	–	–/–/–
*C. f_*17	1,342 bp	PP264351	99.55%	*C. freundii* strain LMG 3246/NR_117752.1	*+*/–/–/–	–	–/–/–
*C. f_*18	1,342 bp	PP264352	99.40%	*C. freundii* ATCC 8090/NR_028894.1	*+*/–/–/–	+	+/–/–
*C. f_*19	1,341 bp	PP264353	98.51%	*C. freundii* ATCC 8090/NR_028894.1	*+*/–/–/–	+	+/–/–
*C.f_*20	1,342 bp	PP264354	99.78%	*C. freundii* strain LMG 3246/NR 117752.1	–/–/–/–	–	–/–/–

**Fig. 2.**
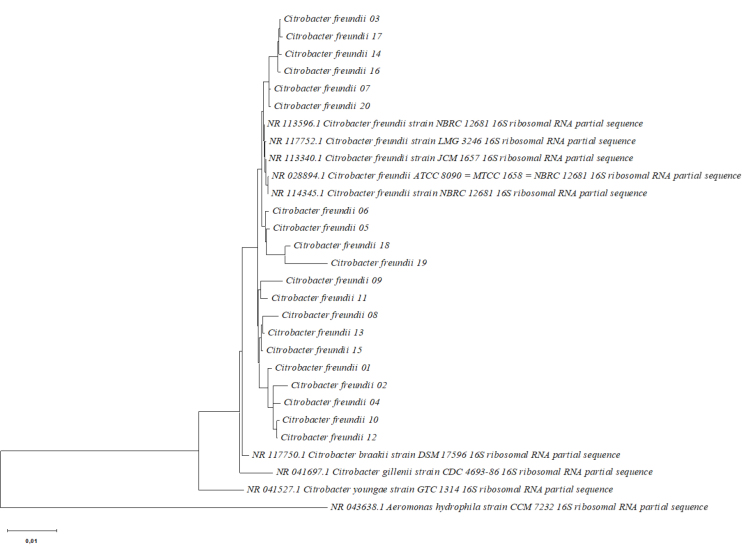
Phylogenetic tree based on the partial *16S rRNA* gene sequences of 20 *Citrobacter freundii* strains isolated from ornamental fish in Poland and 5 *Citrobacter freundii* reference strains

The phylogenetic tree based on *16S rRNA* sequences and drawn using the neighbour-joining method presents a clear division into two main phylogenetic clades (Fig. 2). The branching pattern of the tree demonstrates the existence of genetic relationships between the *Citrobacter* spp. isolates (01–20) and reference strains (NR_028894.1, NR_117752.1, NR_113596.1, NR_113340.1 and NR_114345.1). Taking into account the origin and biochemical properties of the isolates (Table 1 and Supplementary Table 2), the tree also showed genetic heterogeneity and distance within the species (Fig. 2).

### Detection of virulence and drug resistance genes

The virulence gene with the highest prevalence was *viaB*, which was detected in 13 out of 20 isolates (65%) (Table 4). The gene encoding the heat-labile enterotoxin (LT) was detected in only 1 out of 20 isolates (5%) and the gene encoding the heat-stable enterotoxin (ST) was detected in 2 out of 20 isolates (10%, Table 4). Only the presence of the *hlyA* gene encoding the ability to produce α-haemolysins was not confirmed. Detailed information on the occurrence of other genes in isolates is summarised in Table 4.

## Discussion

In our study, 20 isolates of Gram-negative bacteria from 16 different species of ornamental fish were successfully identified to the species level using MALDI-TOF MS as *Citrobacter freundii* bacteria. The usefulness of MALDI methods in identifying *C. freundii* has been demonstrated (20), and the methods have also recently indicated bacteria to be of the *Citrobacter* genus when used on isolates from various environments including sick fish (16). However, the high similarity of the protein profiles of *C. braakii, C. gillenii, C. murliniae, C. rodentium, C. sedlakii, C. werkmanii* and *C. youngae* to those of *C. freundii* leads to these species being collectively referred to as *C. freundii* complex. This results in the need to introduce additional methods to confirm the classification to the species level, as demonstrated by Kolínská *et al*. (20).

The results of biochemical identification were mostly consistent with those previously published on diseased fish (1, 18, 30). Most isolates showed characteristics typical of the species, but deviations were observed in some: these were positive results for ornithine decarboxylation, indole production or amygdaline fermentation and negative results for trisodium citrate utilisation and D-melibiose fermentation. Similar variations in arginine dihydrolase and ornithine decarboxylase activity, indole production and sugar fermentation were previously reported in *C. freundii* isolated from rainbow trout (1). Differences in biochemical profiles may be also due to examined strains’ isolation from different hosts (38). Nevertheless, the biochemical results allowed for reliable identification of strains: 60% of the bacteria had an identification percentage of 99.8/99.9%, confirming that the API-20E system is an important tool for initial identification or as a complementary method.

Fish bacteria of the *Citrobacter* genus can be identified by sequencing the *16S rRNA* gene (10). The sequences of the tested strains showed identity percentages not less than 98.51 to those of reference strains, which in combination with the identification based on the MALDI-TOF MS method and biochemical identification ultimately confirms that they were correctly classified as *C. freundii*. Partial *16S rRNA* gene products were sequenced previously which had been obtained with the same primer pair for *Citrobacter freundii* as was used in the present research (14).

Frederiksen (11) states that the *Citrobacter* genus is naturally sensitive to certain groups of antibiotics: aminoglycosides, sulphonamides, chloramphenicol, trimethoprim, fluoroquinolones, tetracycline, polymyxins, nalidixic acid, nitrofurantoin and fosfomycin. According to the same source, like other representatives of the family *Enterobacteriaceae*, they are insensitive to macrolides (including erythromycin), vancomycin, lincosamides and fusidic acid (11). Additionally, the CLSI (8) states that *C. freundii* is naturally resistant to ampicillin. In our study, the isolates tested showed the highest resistance to ciprofloxacin (65% were resistant), tetracycline (60%), nalidixic acid (60%) and doxycycline (50%). Similar rates of non-susceptibility to these antibiotic groups among *C. freundii* have previously been reported (7). In contrast, the isolates were the most susceptible to carbapenems, *i.e*. imipenem and meropenem (90% and 95% of strains were susceptible), the aminoglycoside gentamicin (effective against 95%) and the combination of an antibiotic and a beta-lactamase inhibitor which is piperacillin-tazobactam (not resisted by 95%). These results are also in agreement with previously published findings (24). The appearance in some strains of insusceptibility to sulfamethoxazole-trimethoprim (40% of isolates were resistant) may be surprising, as it has recently been described as an effective agent against *C. freundii* isolated from ornamental fish (29). The analysis of antibiotic sensitivity also included calculation of the MAR index for each isolate, as the ratio of the number of antibiotics to which it showed resistance to the number of all antibiotics used in the test (21). The MAR index ranged from 0.13 to 0.67. For 16 strains of *C. freundii* (80%), it was equal to or higher than 0.2. The MAR index is a useful tool for assessing health risk and detecting animal isolates that have developed resistance in areas of frequent use of antibiotics. Specifically, a result >0.2 indicates a high-risk source of contamination with antibiotic-resistant microorganisms (21).

The tetracycline class of antibiotics, exemplified by oxytetracycline and tetracycline, are among the most frequently used antibiotics in aquaculture around the world (13). Intensive use of antimicrobials on food fish and ornamental fish may select for highly resistant bacterial strains and promote the horizontal transfer of antibiotic resistance genes between bacteria. Tetracycline resistance genes were detected in representatives of the *Citrobacter* genus using PCR (10, 26). Our research demonstrated that genetic resistance to tetracycline antibiotics was also widespread among samples from ornamental fish from which *C. freundii* was isolated. In this study we focused on the *tetA, tetB* and *tetG* genes, which were present in 40%, 10% and 40% of the isolates, respectively.

It is assumed that the resistance of microorganisms to antibiotics may be determined by genetic information encoded in the circular bacterial chromosome and/or mobile genetic elements, such as plasmids, integrons and transposons. Integrons are divided into classes 1, 2 and 3 based on differences in the structures of the integrase genes. Class 1 integrons are the most frequently detected type in Gram-negative bacteria (33). This study has provided genetic evidence of the presence of integron-encoding genes in *C. freundii* isolated from diseased ornamental fish, and these genes’ presence may contribute to the multidrug resistance of these strains.

ViaB is known as a virulence factor of *C. freundii* (6). The presence of the capsular polysaccharide virulence antigen (Vi) provides resistance to the bactericidal action of the complement system and impedes phagocytosis, thus helping the bacteria evade the host’s natural immune response during infection. Three distinct chromosomal loci are responsible for its expression: *viaA, viaB* and *ompB* (6). It was also present in the strains tested in the present study as the most common virulence factor.

Another virulence factor which can be propagated in strains of *C. freundii* is the heat-labile enterotoxin (LT) typical of *Escherichia coli* (27). Studies have shown that *E. coli* bacteria carrying plasmids with the gene encoding LT can transmit it *via* horizontal gene transfer to other bacteria such as *Klebsiella pneumoniae,Shigella sonnei, Salmonella typhimurium, Enterobacter cloacae* or *C. freundii*. Consequently, these other bacteria acquire enterotoxigenic properties (27). The presence of LT genes in *C. freundii* strains has previously been confirmed in both clinical isolates (6) and isolates from animal samples (37) at higher rates than in the study referred to above where it was experimentally introduced.

In addition to heat-labile toxins, genes responsible for encoding the heat-stable enterotoxin (ST) may be detected in *C. freundii* genomes. These are a group of small proteins that bind to receptors on the surface of the intestines of mammals and therefore often cause diarrhoea in both humans and animals (25). Heat-stable enterotoxins are classified as STa (or STI) or STb (STII). The former can occur in two genetically different forms: STp and STh (25). The group of STa toxins also includes M-ST toxins from *Vibrio mimicus* and C-ST from *C. freundii* (25). The results obtained in our experiment indicate lower carriage than those in the studies of Bunyan (6) and Sekhi and Al-Samarraae (37), in which this gene was present in *C. freundii* with frequencies of 50% and 23.5%, respectively.

Other virulence factors produced by numerous species of Gram-negative bacteria pathogenic to humans and animals are extracellular toxic proteins, specifically haemolysins (43). Their activity leads to the lysis of red blood cells through pore formation. Genes associated with the production and extracellular secretion of haemolysins in *Escherichia coli* are located in the hlyCABD operon. It is worth noting that the *hlyA* gene, which encodes a 110 kDa inactive precursor protein of the mature toxin called pro-HlyA, is present in most uropathogenic *E. coli* strains (28). The presence of haemolysins in fish isolates, especially from the *Vibrio* genus, has also been investigated (34). Although studies indicate the potential occurrence of haemolysins in *C. freundii* originating in fish (4), we were unable to obtain amplification products for any of the strains analysed. The results of genetic tests using the PCR method coincided with the phenotypic testing of the ability to produce haemolysins, performed on blood agar plates.

## Conclusion

The study has shown that *C. freundii* strains are present in the aquarium environment and can be isolated from tissues of diseased ornamental fish. The species identification of all isolates tested was successfully confirmed by three methods: initially by MALDI-TOF MS and then by genetic and biochemical methods. The study of phenotypic profiles of antibiotic sensitivity suggests that diseased ornamental fish may be a reservoir of MDR *C. freundii*. Our work showed that isolates from this environment carry various virulence genes (*viaB, LT* and *STp*) and antibiotic resistance genes (*tetA, tetB* and *tetG*). They may also be a source of class-1 integron–encoding genes associated with the spread of multidrug resistance among aquarium fish. Therefore, further monitoring of *C. freundii* in this environment is needed.

## Supplementary Material

Supplementary Material Details

Supplementary Material Details
