# Supplementary material for: Occurrence of virulence factors and antimicrobial susceptibility of Citrobacter freundii isolated from diseased ornamental fish in Poland
Source: J Vet Res. 2025 Mar 25;69(1):17–26. doi: 10.2478/jvetres-2025-0017 (PMC11936085; doi:10.2478/jvetres-2025-0017)
Supplement: Supplementary file 1 — Supplementary Material Details [file jvetres-2025-0017_sm1.pdf]

Supplementary Table 1. Antibiotic resistance of *Citrobacter* species isolated from freshwater and ornamental fish

Antibiotic	01	02	03	04	05	06	07	08	09	10	11	12	13	14	15	16	17	18	19	20
Penicillins																				
AM (10 µg)	R	R	R	R	R	R	R	R	R	R	R	R	R	R	R	R	R	R	R	R
TZP (110 µg)	S	S	S	S	S	I	S	S	S	S	S	S	S	S	S	S	S	S	S	S
Cephalosporins																				
CTX (30 µg)	I	S	R	S	I	I	S	I	S	S	S	R	I	I	S	S	S	I	S	S
Carbapenems																				
IPM (10 µg)	S	S	S	S	S	S	S	S	I	S	S	S	S	S	S	S	S	S	R	S
MEM (10 µg)	S	I	S	S	S	S	S	S	S	S	S	S	S	S	S	S	S	S	S	S
Aminoglycosides																				
CN (10 µg)	S	I	S	S	S	S	S	S	S	S	S	S	S	S	S	S	S	S	S	S
AK (30 µg)	S	S	S	I	S	S	S	S	S	S	S	I	S	I	S	S	S	R	R	S
K (30 µg)	I	I	S	R	R	I	S	I	S	R	S	R	R	I	S	I	I	I	I	S
S (10 µg)	S	S	I	R	S	R	R	R	I	R	R	R	I	I	R	I	I	I	I	R
Tetracyclines																				
TE (30 µg)	R	R	S	R	R	R	S	R	R	R	R	R	S	S	S	S	S	R	R	S
DO (30 µg)	R	R	S	R	R	R	S	R	R	R	R	R	S	I	S	S	S	I	I	S
Quinolones																				
CIP (5 µg)	R	R	I	R	R	R	I	R	S	R	I	R	I	R	I	R	R	R	R	I
NA (30 µg)	R	I	I	R	R	R	S	R	S	R	I	R	S	R	S	R	R	R	R	S
Sulphonamides																				
SXT (25 µg)	R	S	S	R	R	R	S	S	S	R	S	R	S	S	S	S	S	R	R	S
Phenicols																				
C (30 µg)	R	S	S	R	R	R	S	R	S	R	S	R	S	S	S	S	S	R	R	S
MAR index	0.47	0.27	0.13	0.60	0.53	0.53	0.13	0.47	0.20	0.60	0.27	0.67	0.13	0.20	0.13	0.20	0.20	0.47	0.53	0.13

AM – ampicillin; TZP – piperacillin-tazobactam; CTX – cefotaxime; IPM – imipenem; MEM – meropenem; CN – gentamicin; AK – amikacin; K – kanamycin; S – streptomycin; TE – tetracycline; DO – doxycycline; CIP – ciprofloxacin; NA – nalidixic acid; SXT – sulfamethoxazole-trimethoprim; C – chloramphenicol; MAR – multiple antimicrobial resistance; 01–20 – numbers of the tested *Citrobacter freundii* isolates; R – resistant species; I – intermediate species; S – susceptible species
