# Supplementary material for: Occurrence of virulence factors and antimicrobial susceptibility of Citrobacter freundii isolated from diseased ornamental fish in Poland
Source: J Vet Res. 2025 Mar 25;69(1):17–26. doi: 10.2478/jvetres-2025-0017 (PMC11936085; doi:10.2478/jvetres-2025-0017)
Supplement: Supplementary file 2 — Supplementary Material Details [file jvetres-2025-0017_sm2.pdf]

Supplementary Table 2. Phenotypical characterisation and API 20E test results of *Citrobacter freundii* (n = 20) isolated from ornamental fish

	*C.f*_01	*C.f*_02	*C.f*_03	*C.f*_04	*C.f*_05	*C.f*_06	*C.f*_07	*C.f*_08	*C.f*_09	*C.f*_10
Gram reaction	−	−	−	−	−	−	−	−	−	−
Shape	rods	rods	rods	rods	rods	rods	rods	rods	rods	rods
Motility – swimming(ability/z.d)	+/47	+/34	+/51	+/56	+/54	+/52	+/59	−/9	+/42	+/71
Oxidase reaction	−	−	−	−	−	−	−	−	−	−
Haemolytic test	γ–haem.	γ–haem.	γ–haem.	γ–haem.	γ–haem.	γ–haem.	γ–haem.	γ–haem.	γ–haem.	γ–haem.
Growing on MacConkey agar	lac+	lac+	lac+	lac+	lac+	lac+	lac+	lac+	lac+	lac+
ONPG test (β-galactosidase)	+	+	+	+	+	+	+	+	+	+
Arginine dihydrolase	−	+	−	+	−	+	−	+	+	+
Lysine decarboxylase	−	−	−	−	−	−	−	−	−	−
Ornithine decarboxylase	−	+	−	+	−	+	−	+	+	+
Trisodium citrate utilisation	−	+	+	+	+	+	+	−	+	+
H,S production	+	+	+	+	+	+	+	+	+	+
Urease activity	−	−	−	−	−	−	−	−	−	−
Deamination of tryptophan	−	−	−	−	−	−	−	−	−	−
Indole production	−	+	−	+	−	+	−	−	−	+
Acetoin production (VP test)	−	−	−	−	−	−	−	−	−	−
Gelatin liquefaction	−	−	−	−	−	−	−	−	−	−
D-glucose fermentation	+	+	+	+	+	+	+	+	+	+
D-mannitol fermentation	+	+	+	+	+	+	+	+	+	+
Inositol fermentation	−	−	−	−	−	−	−	−	−	−
D-sorbitol fermentation	+	+	+	+	+	+	+	+	+	+
L-rhamnose fermentation	+	+	+	+	+	+	+	+	+	+
D-saccharose fermentation	+	+	+	+	+	+	+	+	+	+
D-melibiose fermentation	+	+	+	+	+	+	+	−	+	+
Amygdaline fermentation	−	−	−	−	+	−	−	−	−	−
L-arabinose fermentation	+	+	+	+	+	+	+	+	+	+
API-20E code number	1 404 572	3 744 572	1 604 572	3 744 572	1 604 573	3 744 572	1 604 572	3 504 532	3 704 572	3 744 572
API-20E identification	*C. freundii*	*C. freundii*	*C. freundii*	*C. freundii*	*C. freundii*	*C. freundii*	*C. freundii*	*C. freundii*	*C. freundii*	*C. freundii*
API-20E % identification	99.9	62.8	99.9	62.8	99.8	62.8	99.9	59.6	70.3	62.8

	*C.f*_11	*C.f*_12	*C.f*_13	*C.f*_14	*C.f*_15	*C.f*_16	*C.f*_17	*C.f*_18	*C.f*_19	*C.f*_20
Gram reaction	−	−	−	−	−	−	−	−	−	−
Shape	rods	rods	rods	rods	rods	rods	rods	rods	rods	rods
Motility – swimming(ability/z.d)	+/40	+/35	+/32	+/52	+/59	+/53	+/37	+/54	+/30	+/34
Oxidase reaction	−	−	−	−	−	−	−	−	−	−

Haemolytic test	γ–haem.	γ–haem.	γ–haem.	γ–haem.	γ–haem.	γ–haem.	γ–haem.	γ–haem.	γ–haem.	γ–haem.
Growing on MacConkey agar	lac+	lac+	lac+	lac+	lac+	lac+	lac+	lac+	lac+	lac+
ONPG test (β-galactosidase)	+	+	+	+	+	+	+	+	+	+
Arginine dihydrolase	−	+	−	+	−	+	+	+	+	−
Lysine decarboxylase	−	−	−	−	−	−	−	−	−	−
Ornithine decarboxylase	−	+	−	−	−	−	−	−	−	−
Trisodium citrate utilisation	+	+	+	+	+	+	+	+	+	+
H,S production	+	+	+	+	+	+	+	+	+	+
Urease activity	−	−	−	−	−	−	−	−	−	−
Deamination of tryptophan	−	−	−	−	−	−	−	−	−	−
Indole production	−	+	−	−	−	−	−	−	−	−
Acetoin production (VP test)	−	−	−	−	−	−	−	−	−	−
Gelatin liquefaction	−	−	−	−	−	−	−	−	−	−
D-glucose fermentation	+	+	+	+	+	+	+	+	+	+
D-mannitol fermentation	+	+	+	+	+	+	+	+	+	+
Inositol fermentation	−	−	−	−	−	−	−	−	−	−
D-sorbitol fermentation	+	+	+	+	+	+	+	+	+	+
L-rhamnose fermentation	+	+	+	+	+	+	+	+	+	+
D-saccharose fermentation	+	+	+	+	+	+	+	+	+	+
D-melibiose fermentation	+	+	+	−	+	+	+	+	+	+
Amygdaline fermentation	−	−	+	−	+	−	−	−	−	−
L-arabinose fermentation	+	+	+	+	+	+	+	+	+	+
API-20E code number	1 604 572	3 744 572	1 604 573	3 604 532	1 604 573	3 604 572	3 604 572	3 604 572	3 604 572	1 604 572
API-20E identification	*C. freundii*	*C. freundii*	*C. freundii*	*C. freundii*	*C. freundii*	*C. freundii*	*C. freundii*	*C. freundii*	*C. freundii*	*C. freundii*
API-20E % identification	99.9	62.8	99.8	80.2	99.8	99.9	99.9	99.9	99.9	99.9

ONPG – o-nitrophenyl-β-D-galactopyranoside; VP – Voges–Proskauer; + – positives result; − – negative result; z.d – zone diameter in mm; γ-haem – type of haemolysis; lac+ – lactose fermentation
